# Editorial: Host-microbiota immuno-interactions for personalized microbial therapeutics, volume II

**DOI:** 10.3389/fimmu.2026.1906261

**Published:** 2026-07-07

**Authors:** Shashank Gupta, Sunil Kumar Raghav, Nar Singh Chauhan

**Affiliations:** 1 School of Medical Sciences, Faculty of Medicine and Health, Örebro University, Örebro, Sweden; 2 Immuno-genomics and Systems Biology Laboratory, Institute of Life Sciences, Bhubaneswar, Orissa, India; 3 Department of Biochemistry, Maharshi Dayanand University, Rohtak, Haryana, India

**Keywords:** gut microbiota crosstalk, host microbiota interactions, human microbiota, microbial therapeutics, microbiota immune interactions, microbiota therapeutics, personalized therapy

## Introduction

The human gut microbiota, consisting of trillions of microorganisms, is regarded as a complex organ-like ecosystem that emerges through a highly selective colonization process and exerts a profound effects on host health (1). Microorganisms inhabiting the gut mucosa continuously engage with host immune system through metabolic exchange (2, 3). These microbial cues shape both innate and adaptive immune responses, maintaining a delicate balance between immune tolerance and inflammation while simultaneously influencing the composition and function of the gut microbiota (4). These host–microbiota immune-interactions are fundamental to maintaining health and physiological homeostasis (3). Importantly, this crosstalk is highly specific, and individual microbes can exert unique and measurable effects on host immune function (5). Colonization with segmented filamentous bacteria promotes the development of intestinal Th17 cells (6), whereas *Clostridia* species belonging to clusters IV, XIVa, and XVIII increase the number of Foxp3^+^ regulatory T cells in the colon, particularly in environments rich in TGF-β (7). These immunomodulatory effects are mediated by microbial metabolites such as short-chain fatty acids, secondary bile acids, and polyamines. These microbial metabolites regulate immune cell differentiation (8), cytokine production (9), and epithelial barrier integrity (10). Beyond metabolites, microbial structural molecules also influence immune responses. Sphingolipids produced by *Bacteroides fragilis* modulate invariant natural killer T cell development during the neonatal period, thereby providing long-term protection against intestinal inflammation and colitis (11).

The host–microbiota immune-interactions begin early and extend far beyond the gut (12). Early-life microbial colonization is critical for educating the immune system, shaping immune competence and disease susceptibility throughout life (13). Continuous signaling from commensal microbes helps to establish the activation threshold of systemic immune responses, thereby influencing the host’s ability to resist infections and maintain immune homeostasis (14). Conversely, disruption of this finely tuned host–microbiota–immune equilibrium, often referred to as dysbiosis, can contribute to the onset of pathological conditions.

The growing understanding of host-microbiota immune-interactions provides a mechanistic bridge between correlation and clinically actionable biology. By understanding how specific microbes and their products modulate immune pathways, researchers can design targeted interventions. Such insights form the foundation for personalized microbial therapeutics, including microbiome-guided probiotics and prebiotics, engineered live biotherapeutic products, targeted enhancement of mucosal barrier function and IgA responses, and precision microbiome modulation strategies tailored to an individual’s immune and microbial profile. Research unveiling microbe–immune interactions provides a mechanistic link from correlation and causation, paving the way for clinically actionable therapies (15). In sum, the host–microbiota immune axis offers a rational framework for precision medicine.

## Research and review articles

A central theme emerging from this research topic is the role of microbial dysbiosis as both a driver of disease pathogenesis and a source of clinically relevant biomarkers. Yang et al. investigated gut microbiota alterations in infants and young children with severe pneumonia-associated sepsis and identified distinct microbial signatures characterized by reduced microbial diversity, increased *Enterobacteriaceae* abundance, and depletion of beneficial short-chain fatty acid-producing taxa. Their findings suggest that gut microbial profiles may serve as early biomarkers for risk stratification in critically ill pediatric patients. Similarly, Zhou et al. examined gut microbiota alterations in patients with HBV-related cirrhosis and spontaneous bacterial peritonitis (SBP). The authors developed a novel microbiota-derived index (SBP-MI) and integrated it with clinical parameters to establish an SBP risk prediction model with strong predictive performance. This study highlights the translational potential of microbiome-based tools for predicting disease progression and improving clinical management.

Expanding the diagnostic potential of microbiome research, Sun et al. identified characteristic intestinal microbial signatures associated with hepatocellular carcinoma across different stages of chronic liver disease. By integrating microbiota profiles with clinical indicators, the authors developed a predictive model capable of distinguishing patients at increased risk for hepatocellular carcinoma, supporting the use of microbiome-informed approaches in cancer surveillance.

The growing integration of advanced computational and multi-omics technologies into microbiome research is exemplified by the work of Jiang et al. who combined microbiome sequencing, metabolomics, Mendelian randomization analysis, and machine learning approaches to investigate diabetic kidney disease. Their integrated analysis revealed microbiota-metabolite-host interaction networks and identified candidate biomarkers capable of distinguishing diabetic kidney disease from type 2 diabetes with high accuracy. These findings illustrate how systems biology approaches can facilitate the development of precision diagnostic strategies.

Several studies in this Research Topic focused on elucidating mechanisms by which microbial communities influence immune responses. Chen et al. reported that pasteurized *Akkermansia muciniphila* alleviated obesity-induced bone loss by promoting regulatory T-cell differentiation through the Nr4a1-dependent pathway. Their observations revealed a previously unrecognized microbiota–immune–bone axis and suggested that postbiotic approaches may represent promising therapeutic strategies for metabolic bone disorders. In another study involving *Akkermansia muciniphila*, Kim et al. investigated the effects of this next-generation probiotic during SARS-CoV-2 infection. Using a transgenic mouse model, the authors showed that prophylactic administration of *A. muciniphila* enhanced lung-resident antiviral immunity, promoted tissue-resident memory T-cell responses, and improved pulmonary pathology through modulation of the gut–lung axis. These findings further highlight the systemic immunomodulatory capacity of gut microbes and their potential therapeutic application in respiratory viral infections.

The complexity of microbiota-based interventions was further explored by Smout et al. who examined microbiota obtained from probiotic-treated preterm infants using a microbiota-humanized mouse model. Their study demonstrated that probiotic-conditioned microbial communities influenced immune development and altered responses to enteropathogenic bacterial challenge. Importantly, the findings emphasized that microbiota-targeted interventions may have context-dependent effects and should be carefully evaluated in vulnerable populations such as preterm infants. Restoration of microbial homeostasis as a therapeutic strategy was also addressed by (Lohani et al. ). Using HIV-infected double humanized BLT mice receiving antiretroviral therapy, the authors demonstrated that fecal microbiota transplantation reduced systemic inflammation, modulated intestinal gene expression, and improved markers associated with microbial translocation. Their findings support the potential use of microbiota restoration therapies as adjunctive approaches for managing chronic immune activation in people living with HIV. Mucosal immunity represents another important aspect of host–microbiota interactions. Zhou et al. developed an innovative Alhagi honey polysaccharide-aluminum Pickering emulsion adjuvant capable of enhancing intestinal mucosal immunity. The adjuvant promoted intestinal IgA production while simultaneously stimulating systemic immune responses, highlighting novel opportunities for microbiome-inspired vaccine strategies and mucosal immune modulation. Further mechanistic insight into microbial regulation of mucosal immunity was provided by Bernard et al., who investigated microbial sensing through the non-canonical inflammasome pathway. Their work demonstrated that epithelial sensing of bacterial lipopolysaccharide via caspase-4-dependent signaling promoted IL-33 release and influenced type 2 immune responses in the airway. These findings provide a mechanistic framework linking microbial recognition pathways with allergic inflammation and asthma pathogenesis.

Studies also highlighted the central role of host–microbiota–immune interactions in maintaining health and driving disease across diverse anatomical sites. Lv et al. summarized the contributions of dysbiosis, epithelial barrier dysfunction, and aberrant immune activation to the development of chronic inflammatory and neoplastic oral diseases. Sîrbu et al. reviewed current knowledge on the pediatric nasal microbiome and its association with chronic ear, nose, and throat disorders. This article emphasizes the influence of early-life factors such as mode of delivery, breastfeeding, antibiotic exposure, environmental pollution, and passive smoking on microbial colonization and immune development. Chen et al. explored the critical relationship between gut microbiota dysbiosis and the development of immune-mediated liver diseases. The authors highlighted the role gut microbes, microbial metabolites, and other biological mediators in regulating gut-liver axis. This review also underscores the potential of restoring microbial homeostasis to manage chronic liver inflammation and improve patient outcomes. Wang et al. provided preliminary evidence that modulation of immune signaling through tofacitinib improves clinical outcomes in ankylosing spondylitis. Their findings further suggest that tofacitinib may influence gut microbial composition, supporting the existence of a gut–joint axis. Zhang et al. reported a case of severe *Escherichia coli*-induced bullous erysipelas, illustrating the clinical consequences of immune dysregulation in immunocompromised individuals. Collectively, these studies underscore that disruptions in microbial ecology and immune regulation significantly influence disease pathogenesis, progression, and therapeutic responsiveness.

Collectively, the studies presented in this volume demonstrate the broad impact of host–microbiota immune-interactions across diverse disease settings, including infectious diseases, metabolic disorders, liver diseases, respiratory conditions, pediatric illnesses, and cancer. Importantly, these contributions move the field beyond descriptive analyses of microbial composition toward mechanistic understanding, biomarker discovery, and development of personalized microbial therapeutics.
